# Editorial: Peptidases as a therapeutic target in anti-cancer management

**DOI:** 10.3389/fphar.2024.1394018

**Published:** 2024-04-11

**Authors:** Ana Mitrović, Roberta E. Burden, Adaleta Softič, Nežka Kavčič

**Affiliations:** ^1^ Department of Biotechnology, Jožef Stefan Institute, Ljubljana, Slovenia; ^2^ Faculty of Pharmacy, University of Ljubljana, Ljubljana, Slovenia; ^3^ School of Pharmacy, Medical Biology Centre, Queen’s University Belfast, Belfast, United Kingdom; ^4^ University of Tuzla, Tuzla, Bosnia and Herzegovina; ^5^ International Centre for Genetic Engineering and Biotechnology, Trieste, Italy

**Keywords:** peptidases, cancer, anti-tumor treatment, cathepsins, ADAM17, ubiquitin specific protease, sentrin specific-protease

Peptidases are a large family of proteolytic enzymes that catalyze the hydrolysis of peptide bonds. They account for about 2% of the total protein-coding genome (Rawlings et al., 2018). Peptidases are localized extracellularly or intracellularly in the cytoplasm, the nucleus or packaged into lysosomal/endosomal vesicles. Peptidases have important physiological functions in a number of biological processes where they act either as degradative enzymes or as regulatory enzymes that alter peptide function after cleavage of one or more specific peptide bonds.

The Research Topic “*Peptidases as a therapeutic target in anti-cancer management*” is dedicated to proteolytic enzymes and their potential as a target for cancer treatment. Under physiological conditions, the activity of peptidases is tightly regulated by a variety of factors. Dysregulation of peptidase activity is associated with a number of pathological processes, including cancer. Due to their important role in cancer progression, peptidases have been identified as promising therapeutic targets for the development of new therapeutic approaches that could improve cancer treatment. This Research Topic is dedicated to shedding light on peptidases as a therapeutic target in the fight against cancer. The papers focus either on the identification of new peptidase inhibitors or on the mechanisms of peptidases in cancer progression and their potential to be used as therapeutic targets. The peptidases from different families and with different mechanisms of action are presented.

Chi et al. present the development of new small-molecule ADAM17 inhibitors. A disintegrin and metallopeptidase 17 (ADAM17) belongs to the ADAM family of disintegrins and metallopeptidase, which contributes to the activation of the Notch pathway in cancer progression by cleavage of the Notch protein/receptor and releasing the Notch intracellular domain (NICD). Released NICD mediates survival of cancer cells and increases their resistance to antitumor treatment. Therefore, targeting ADAM17 represents an interesting approach to inhibit the cleavage and activation of the Notch pathway, to increase the sensitivity to antitumor treatment and to overcome drug resistance of cancer cells (Meurette and Mehlen, 2018; Calligaris et al., 2021; Zhou et al., 2022). Chi et al. used molecular docking, virtual screening and structural fragment design of the lead compound to identify compound **2b** as a novel potent selective small-molecule inhibitor of ADAM17 and evaluate its effect on the treatment of non-small cell lung cancer (NSCLC). They showed that compound **2b** inhibited the cleavage and activation of the Notch pathway and increased the sensitivity of NSCLC cells to antitumor drugs.

Attractive targets for improvement of antitumor treatment are lysosomal cysteine cathepsins. Smyth et al. described the evaluation of variable new antigen receptors (vNARs) as a new strategy for targeting the lysosomal cysteine peptidase cathepsin S (CatS). vNARs are the major component of the adaptive immune system in sharks and the smallest naturally occurring single-chain binding domains in vertebrates. vNARs differ in their properties and are reported to be stable within cells, making them attractive candidates as inhibitors (Barelle and Porter, 2015). The authors developed and characterized a panel of novel vNARs targeting pro-CatS obtained by a phage display panning process. The lead vNAR binders showed binding to pro-CatS and affected the activity of the enzyme in a dose-dependent manner. The identified vNARs were shown to prevent the conversion of the inactive proform to the mature active form of CatS, representing a novel mechanism for its inhibition. Furthermore, vNAR clones were able to impair cell invasion in the tumor cell invasion assay.

In the second study on lysosomal cysteine cathepsins, Sereeongsaeng et al. investigated cathepsin V (CatV) and its regulation of cell cycle and histone stability. The results support CatV as a new therapeutic target in targeting ER + breast cancer. The study showed that CatV has an impact on the proliferation of breast cancer cells, revealing that nuclear CatV is required for cell cycle progression and histone stability in breast cancer cells. The effect of CatV on cell cycle progression and proliferation was demonstrated by the regulation of histone H3 and H4 protein expression through regulation of their chaperone sNASP. Taken together, the results show that targeting CatV within the nucleus could be an interesting therapeutic strategy to impair cell cycle progression in breast cancer.

Another class of proteolytic enzymes involved in cancer progression are the deubiquitinating enzymes (DUBs), a family of enzymes that mediate the removal of ubiquitin molecules from target proteins and have a critical role in tumor progression. Among other cancers, DUBs play a critical role in glioblastoma multiforme (GBM), where they have been reported as potential therapeutic targets due to their multifunctional involvement in tumor progression (Young et al., 2019). Li et al. showed ubiquitin specific protease 5 (USP5) plays a crucial role in tumorigenesis and progression of GBM by stabilizing cyclin D1. USP5 is a ubiquitin specific protease that belongs to the subclass of DUBs. It has been shown that USP5 directly interacts with cyclin D1 and reduces its K48-linked polyubiqutination. Overexpression of USP5 significantly prolonged the half-life of cyclin D1, while knockout of USP5 decreased the protein level of cyclin D1 and led to suppression of proliferation, colony formation of GBM cells and inhibition of tumor growth *in vivo*.

In the last study of this Research Topic, Taghvaei et al. introduced the sestrin specific-protease 1 (SENP1), a protein involved in deSUMOylation, as a protease overexpressed in various types of cancer and as possible biomarker and molecular target. The authors evaluated SENP1 expression, function and molecular mechanisms in carcinogenesis for the prognosis and treatment of cancer using computational biology methods. For SENP1, they showed a role in immune infiltration and as an expression marker, indicating the possibility that SENP1 can be introduced as a target for tumor immunotherapy. Furthermore, SENP1 was shown to correlate with sensitivity and resistance to antitumor treatment. In addition, the authors also presented drugs used in clinical practice that can inhibit SENP1.

Collectively, the articles in this Research Topic highlight the importance of different peptidase families in the development and progression of cancer as well as their enormous potential to be used as therapeutic targets in the fight against cancer. Further research in this field should therefore aim at a better understanding of the mechanisms of peptidases in cancer progression and the development of new therapeutic approaches that could improve the treatment of the disease.
